# The association between the number of oocytes retrieved and cumulative live birth rate in different female age strata

**DOI:** 10.1038/s41598-023-41842-7

**Published:** 2023-09-04

**Authors:** Peixin Wang, Chenqiong Zhao, Wen Xu, Xiaoying Jin, Songying Zhang, Haiyan Zhu

**Affiliations:** 1 Assisted Reproduction Unit, Department of Obstetrics and Gynecology, Sir Run Run Shaw Hospital, School of Medicine, Zhejiang University, Hangzhou, China; 2 Key Laboratory of Reproductive Dysfunction Management of Zhejiang Province, Zhejiang Provincial Clinical Research Center for Obstetrics and Gynecology, Hangzhou, 310016 China

**Keywords:** Medical research, Outcomes research

## Abstract

To evaluate the association between the number of oocytes retrieved and cumulative live birth rate (CLBR) in different female age strata. 17,931 women undergoing their first IVF/ICSI-ET cycle in the Sir Run Run Shaw Hospital of Zhejiang University were grouped by age (A: ≤ 35 years; B: ≥ 36 years) as well as the number of oocytes retrieved (a: ≤ 5; b:6–9; c:10–14; d: ≥ 15). Multivariate regression analysis was performed to assess the OR of CLBR for the variable ‘age’ and ‘number of oocytes retrieved’. The group ≥ 36 years exhibited lower cumulative pregnancy rates (CPRs) and cumulative live birth rates (CLBRs), which are proportional to the number of oocytes retrieved but opposite to increasing age. Multivariate logistic regression analysis revealed that the age and number of oocytes retrieved remain significant independent predictive factors (P < 0.001). Age and number of oocytes retrieved are two independent factors affecting the CLBR. The discrepancy of the minimum number of oocytes retrieved for patients with different ages to achieve ideal CLBR is instructive for clinical practice. The practice of controlling the stimulation dose is feasible for patients ≤ 35 years who can achieve over 60% CLBR once the number of oocytes obtained is more than 6. However, additional stimulation cycles and accumulation of embryos are necessary for elderly group especially those ≥ 38 years old who need more than 14 oocytes to obtain higher live birth rate.

## Introduction

Controlled ovarian stimulation is an important part of assisted reproductive technologies (ARTs)^1,2^. With the substantial advancement of ART in both clinical and laboratory, the number of freeze–thaw embryo transfer cycle have increased along with the pregnancy rate^3,4^. Clinical or ongoing pregnancy rate, embryo implantation rate and live birth rate per transfer have consuetudinary been used to evaluate the successful rate of ART while the cumulative live birth rate (CLBR) appears to be a better one currently. The CLBR, which mainly refers to the first live birth after using all embryos for an integrated cycle (including fresh and subsequent freeze–thaw cycles), varies synchronously with the number of oocytes retrieved^5,6^. Previous studies have manifested that CLBR can even reach as high as 70% once the oocytes retrieved are more than 25 and with no obvious plateau^7^. Despite the benefit of ART in achieving adequate multiple follicles and providing considerable embryos for selection, the optimal number of oocytes need to retrieve and corresponding probability of live birth in elderly women after each ovulation induction therapy have not been determined.

As the two-child policy fully issued in China since 2015, the demand for ART treatment has been growing steadily among women of advanced reproductive age^8^. An additional 90 million couples in China intend to have a second child according to official estimation, of whom 60% are reported to be older than 35 years, and 50% will be at least 40 years old^8^. Female age becomes the main challenge impacting the success rate of ART. Women ≥ 35 years old have been verified to present a significant decline in the pregnancy rates and live birth rates^9–11^. As is known to all, ovarian function and oocyte quality are all inextricably linked with female age, with the group ≥ 35 years of age certainly experiencing less chance to achieve high number of oocytes retrieved as well as higher risk of developing aneuploidy oocytes and embryos^12–14^. Therefore, challenges that often encountered in clinical practice are as follows: How many oocytes are sufficient to achieve a live birth for women of advanced reproductive age? How many IVF/ICSI cycles are required for the elderly women to increase the probability of live birth?

The purpose of this research was to reveal the relationship between the number of oocytes retrieved and the cumulative live birth rate (CLBR) in different female age strata after categorizing patients by age.

## Results

### Characteristics of basic clinical data

The baseline data of 17,931 women undergoing their first IVF/ICSI cycle were presented in Table1. Comparisons which mainly between patients ≤ 35 years of age and ≥ 36 years of age revealed significant discrepancies in levels of day3 basal FSH, LH and E2, duration of infertility and infertility factors. The group (age ≥ 36 years of age) had significant longer duration of infertility (5.0 ± 4.0 *vs* 3.3 ± 2.3, p < 0.001) but with higher basal FSH (8.4 ± 3.4 vs 7.1 ± 2.6, p < 0.001) and E2(43.0 ± 27.4 vs 41.0 ± 28.2, p < 0.001) levels. In addition, the ovarian stimulation characteristics reported in Table 2 showed that stimulation protocol, duration of stimulation or insemination type all varied strikingly in two age groups.

**Table 1:** Characteristics of basic clinical data.

	≤ 35 years of age	≥ 36 years of age	Statistic	P value	OR (95% CI)
No. of stimulation cycle, n (%)	14,567 (81.2%)	3364 (18.8%)			
Age–the time of oocytes retrieval (meanII ± SD)	29.9 ± 3.0	38.8 ± 2.6	− 172.26	< 0.001	
D3 FSH level (IU/L) (mean ± SD)	7.1 ± 2.6	8.4 ± 3.4	− 18.841	< 0.001	
D3 E2 level (ng/L) (mean ± SD)	41.0 ± 28.2	43.0 ± 27.4	− 3.597	< 0.001	
D3 LH level (IU/L) (mean ± SD)	4.8 ± 4.5	4.3 ± 4.7	4.982	< 0.001	
Infertility factor, n (%)			384.92	< 0.001	
Tubal factor	9384 (64.4%)	2260 (67.2%)			0.884 (0.817–0.958)
Endometriosis	675 (4.6%)	102 (3.0%)			
Ovulatory disorder	630 (4.3%)	49 (1.5%)			
Diminished ovarian reserve	532 (3.7%)	358 (10.6%)			
Male factor	2463 (16.9%)	418 (12.4%)			
Unexplained	883 (6.1%)	177 (5.3%)			
Duration of infertility (years) (mean ± SD)	3.3 ± 2.3	5.0 ± 4.0	− 22.371	< 0.001	
Stimulation protocol, n (%)			1759.592	< 0.001	
Long protocol	8295 (56.9%)	881 (26.2%)			3.727 (3.429–4.052)
Short protocol	1625 (11.2%)	764 (22.7%)			
Ultralong protocol	1380 (9.5%)	241 (7.2%)			
Micro-stimulation protocol	771 (5.3%)	751 (22.3%)			
Others	2496 (17.1%)	727 (21.6%)			
Duration of stimulation (day) (mean ± SD)	9.8 ± 2.0	8.7 ± 2.7	21.851	< 0.001

**Table 2:** Clinical outcomes of fresh cycle and freeze–thaw cycle.

	≤ 35 years of age	≥ 36 years of age	Statistic	P value	OR (95% CI)
No. stimulation cycle, n (%)	14,567 (81.2%)	3364 (18.8%)			
No. of oocytes retrieved (mean ± SD)	10.5 ± 6.5	5.8 ± 4.6	48.902	< 0.001	
No. of embryos can be transferred (mean ± SD)	6.0 ± 4.2	3.5 ± 2.9	32.271	< 0.001	
Use of ICSI, n (%)	4937 (33.9%)	1403 (41.7%)	73.019	< 0.001	0.717 (0.664–0.774)
Fertilization rate (%)					
IVF	70,154/101,362 (69.2%)	8631/12,061 (71.6%)	28.058	< 0.001	0.893 (0.857–0.931)
ICSI	35,589/46,928 (75.8%)	5300/6807 (77.9%)	13.382	< 0.001	0.892 (0.840–0.949)
Good quality embryo rate (%)	49,109/103,765 (47.3%)	6727/13,704 (49.1%)	15.048	< 0.001	0.932 (0.899–0.966)
No. of embryo transfer cycle	18,670	4498			
D3 embryo transfer, n (%)	15,950 (85.4%)	3909 (86.9%)	6.395	0.011	0.884 (0.803–0.973)
Blastocyst transfer, n (%)	2719 (14.6%)	589 (13.1%)			
Thawing cycle, n	16,543	4019			
Thawing cycle transfer, n (%)	16,369/16,543 (98.9%)	3975/4019 (98.9%)	0.057	0.811	1.041 (0.747–1.452)
Good quality embryo transfer rate (%)	22,398/33,788 (66.3%)	4910/7981 (61.5%)	64.861	< 0.001	1.230 (1.169–1.294)
Avg. no. of Blastocyst transfer (mean ± SD)	1.6 ± 0.5	1.6 ± 0.5	0.564	0.571	
Avg. No.D3 embryos, (mean ± SD)	1.9 ± 0.4	1.8 ± 0.5	5.029	< 0.001	
Endometial thickness (mm) (mean ± SD)	9.4 ± 2.7	9.3 ± 2.6	2.527	0.012	
Per.transfer pregnancy rate, n (%)	11,380/18,670 (61.0%)	1795/4498 (39.9%)	654.601	< 0.001	2.351 (2.199–2.512)
Per.transfer embryonic implement rate, n (%)	15,136/33,788 (44.8%)	2192/7981 (27.5%)	798.897	< 0.001	2.143 (2.031–2.261)
Per fresh cyclical live birth rate, n (%)	1420/2301(61.7%)	168/523(32.1%)		< 0.001	3.406 (2.783–4.168)
Per.transfer embryonic live birth rate n (%)	12,582/33,788 (37.2%)	1567/7981 (19.6%)	893.191	< 0.001	2.429 (2.288–2.577)
Spontaneous miscarriages (clinical pregnancy, before 12 weeks), n (%)	965/11,380 (8.5%)	355/1795 (19.6%)		< 0.001	0.379 (0.332–0.433)
Spontaneous miscarriages (clinical pregnancy, after 12 weeks),), n (%)	133/11,380 (1.2%)	37/1795 (2.1%)		0.002	0.553 (0.385–0.796)
Multiple birth rate (%)	2703/10,040 (26.9%)	232/1249 (18.6%)	40.231	< 0.001	1.615 (1.391–1.875)
Male infant rate (%)	5998/12,582 (47.7%)	762/1567 (48.6%)	0.511	0.475	0.962 (0.866–1.069)
Wt.single pregnancy (g), (mean ± SD)	3293.8 ± 525.6	3267.5 ± 538.3	1.543	0.123	
Wt. gemellary pregnancy (g), (mean ± SD)	2444.5 ± 445.0	2455.1 ± 475.2	− 0.34	0.734	
< 37 weeks preterm birth, n (%)	1427/10,040 (14.2%)	172/1249 (13.8%)	0.179	0.673	1.037 (0.875–1.230)
< 34 weeks preterm birth, n (%)	375/10,040 (3.7%)	42/1249 (3.4%)	0.433	0.511	1.115 (0.806–1.542)

### Clinical outcomes of fresh cycle and freeze–thaw cycle

As illustrated in Table 2, the fertilization rate of IVF were higher in the group (women ≥ 36 years of age) (P < 0.01) while the number of oocytes retrieved and good quality embryos rate remained lower compared to other group (women ≤ 35 years of age). In general, there are totally 18,670 embryo transfer cycles in the aged ≤ 35 years group and 4498 in the aged ≥ 36 years group, Day3 embryos were transferred in the majority of embryo transfer cycles. We can observe that the group (age ≥ 36 years of age) had a significantly lower pregnancy rate 39.9% vs. 61.0%; OR: 2.351; 95% CI: 2.199–2.512; P < 0.001) and implantation rate (27.5% vs. 44.8%, OR: 2.143; 95% CI: 2.031–2.261; P < 0.001)compared with the group (age ≤ 35 years of age), which accords with a lower live birth rate (19.6% vs. 37.2%; OR: 2.429; 95% CI: 2.288–2.577; P < 0.001). However, no significant differences in live birth weight and preterm birth rate were found between the two groups.

### The cumulative pregnancy rate and live birth rate.

**Figure 1:**
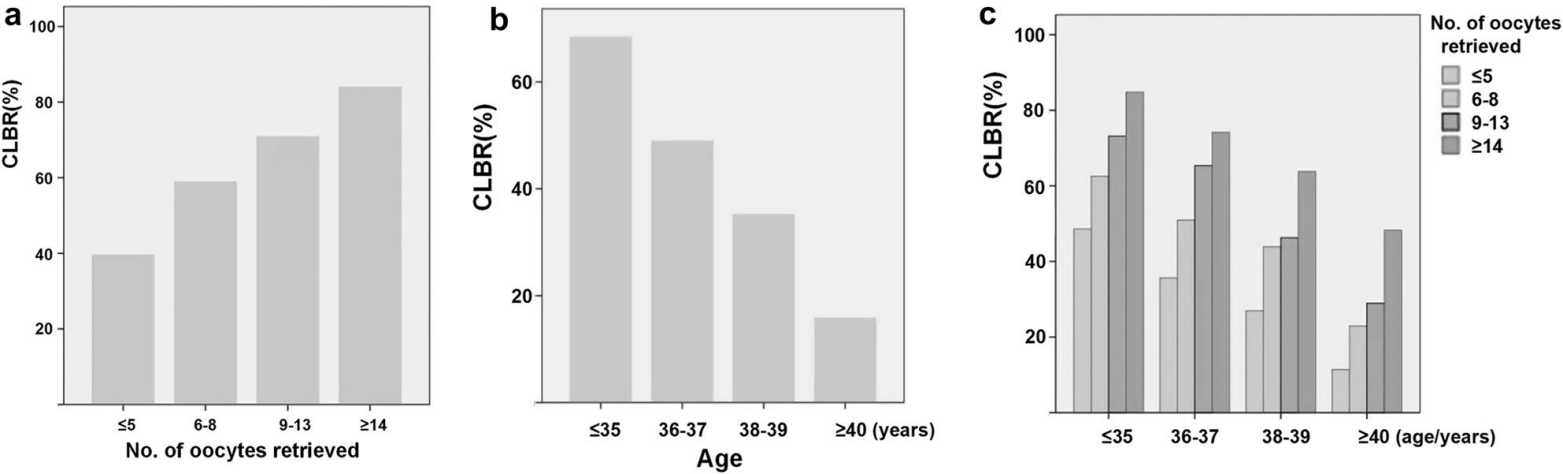
**a** The association between CLBR and the number of oocytes. **b** The association between CLBR and the age. **c** The association between CLBR and the number of oocytes in different female age strata.

**Figure 2:**
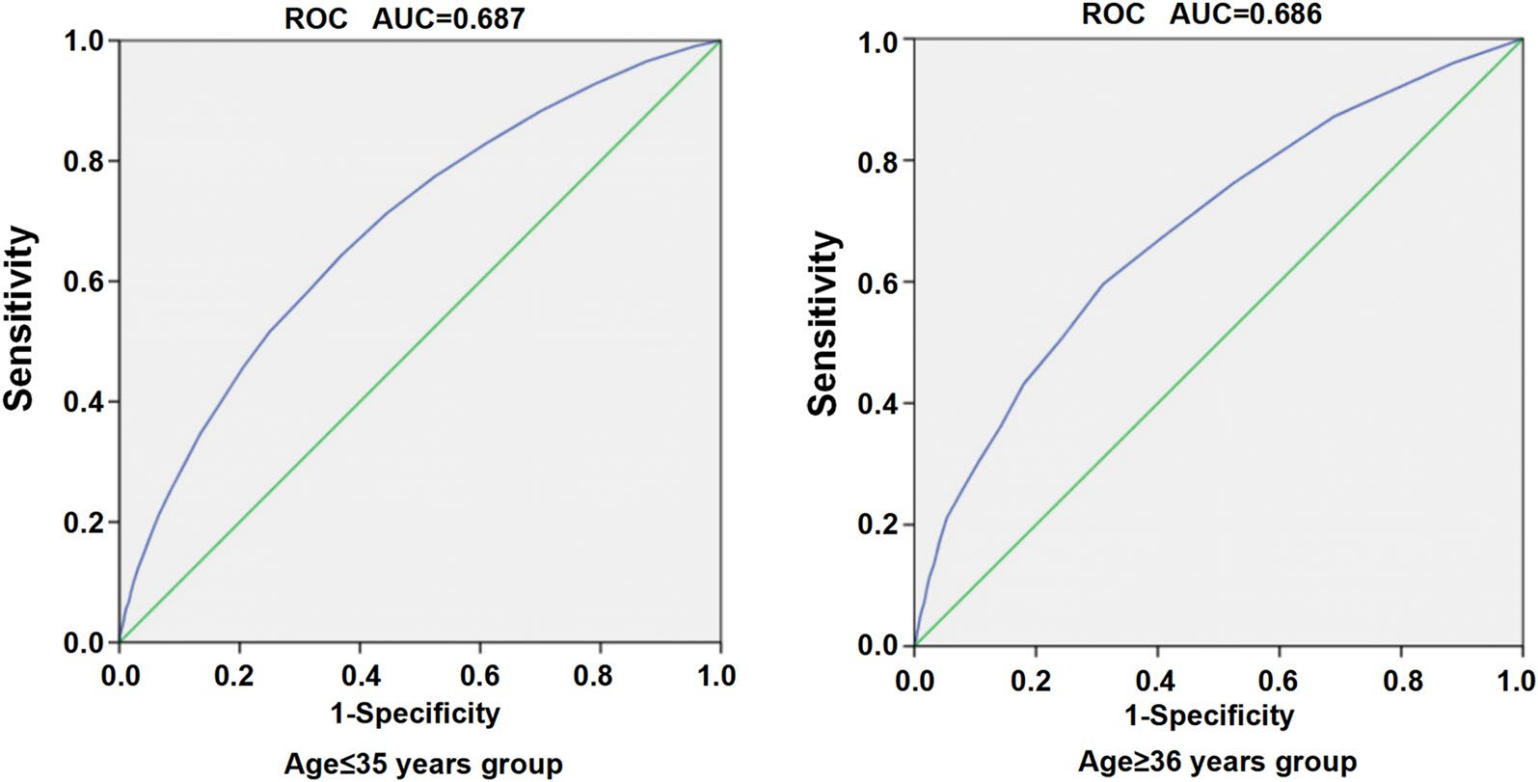
**a** ROC curve in women ≤ 35 years of age. **b** ROC curve in women ≥ 36 years of age.

The cumulative pregnancy rate (CPR) and cumulative live birth rate (CLBR) after one complete IVF/ICSI cycle including fresh and subsequent frozen-thaw cycles from first oocyte retrieval, which were significantly lower in the aged ≥ 36 years group compared with the aged ≤ 35 years group (CPR: 45.0% vs. 74.9%; OR: 3.639; 95% CI: 3.367–3.932; P < 0.001; CLBR: 34.4% vs. 68.3%; OR: 4.125 95% CI: 3.811–4.466; P < 0.001; respectively), as shown in Table 3. Moreover, statistical analysis was performed in different age strata after grouped according to the number of oocytes retrieved. As expected, the CLBR, taking into account fresh and frozen cycles, increased strikingly parallel with the number of oocytes retrieved in specific age strata but decreased with increasing age for a given number of retrieved oocytes (Fig. 1a,b,c). In addition, we performed ROC curves to offer more precise data regarding the optimal number of oocytes required to achieve a cumulative live birth (CLB) between young (≤ 35 years group) and older participants (≥ 36 years of age) (Fig. 2a,b).

**Table 3:** Comparions with the cumulative pregnancy and live birth rate.

	≤ 35 years of age (n = 14,567)	≥ 36 years of age (n = 3364)	Statistic	P value	OR (95% CI)
Cumulative pregnancy rate					
hCG(+) (≥ 50 IU/L), n (%)	11,734/14,567 (80.6%)	1840/3364 (54.7%)	993.183	< 0.001	3.431 (3.169–3.714)
Clinical pregnancy, n (%)	10,905/14,567 (74.9%)	1514/3364 (45.0%)	1144.116	< 0.001	3.639 (3.367–3.932)
Cumulative live birth, n (%)	9957/14,567 (68.3%)	1156/3364 (34.4%)	1339.757	< 0.001	4.125 (3.811–4.466)
Fresh embryo transfer, n (%)	1108/9957 (11.1%)	141/1156 (12.2%)	1.187	0.279	0.901 (0.748–1.087)
Frozen-thawed embryo transfer, n (%)	8849/9957 (88.9%)	1015/1156 (87.8%)	1.187	0.279	1.109 (0.920–1.337)

### Multivariate logistic regression analysis for the cumulative live birth rate.

To control possible confounding variables that might have changed over the study period, a multivariate logistic regression analysis was applied to estimate the OR with SE and 95% CI of the CLBR for variable “age” and “number of oocytes retrieved”. As shown in Table 4, after adjustment for relevant confounders including duration of stimulation, duration of infertility, endometrial thickness, insemination type, fertilization rate, and good quality embryo rate, the age and number of oocytes retrieved remained the independent predictive factors (P < 0.001). The OR of CLBR dropped from 3.695 (95%CI: 2.960–4.611) in the aged ≤ 35 years group to 3.164 (95%CI: 2.563–3.906) and 2.230 (95%CI: 1.785–2.786) in the aged 36–37 years and 38–39 years group respectively. In addition, the fertilization rate, good embryo quality rate and insemination method also have prominent effect on CLBR.

**Table 4:** Multivariate logistic regression analysis for the cumulative live birth rate.

Cumulative live birth rate	OR	SE	95% CI	P value
Age				< 0.001
≤ 35 years of age	3.695	0.113	2.960–4.611	
36–37 years of age	3.164	0.108	2.563–3.906	
38–39 years of age	2.23	0.114	1.785–2.786	
≥ 40 years of age	1			
No.of oocytes retrieved	1.146	0.004	1.138–1.155	< 0.001
Fertilization rate	3.72	0.084	3.720–3.154	< 0.001
Good embryo quality rate	2.713	0.056	2.432–3.027	< 0.001
Insemination method				
IVF	1.095	0.036	1.020–1.174	0.012
ICSI	1			

## Discussion

Results of this study indicated that the CLBR was proportional to the number of retrieved oocytes, and this steady trend was evident in all female age strata. Notably, female age has a negative effect on the relationship between the number of retrieved oocytes and CLBR, with lower CLBR accompanied by increasing age for a given number of retrieved oocytes. Furthermore, older women exhibited a significantly lower LBR and CLBR in their fresh or frozen embryo transfer cycles compared with younger women. The CLBR, which decreased strikingly from 68.3% in the group ≤ 35 years of age to 34.4% in the group ≥ 36 years of age, can even drop to less than 20% in the group ≥ 40 years of age.

Our research is completely consistent with two recent studies investigating the effect of retrieved oocytes numbers as well as frozen embryo transfer on the CLBR^1,7^. Polyzos et al. confirmed that the CLBR could reach as high as 70% once the number of retrieved oocytes was more than 25^7^. However, according to our analysis, the CLBR closing to 80% is possible as long as the retrieved oocytes numbers were more than 25, which can be explained by the discrepancies that CLBs are mainly from freeze–thaw embryo transfers in our study, accounting for 88.9% in the group ≤ 35 years of age and 87.8% in the group ≥ 36 years of age. Since vitrification was introduced into our reproductive center, 97% of the survival rate in embryo cryopreservation, nearly 60% of the pregnancy rate in freezing–thawing embryo transfer, 35% of the embryo implantation rate and 29% of the live birth rate per embryo were achieved among approximately 6,000 freezing–thawing cycles every year^15^.

In the past decades, researches have been proposed to explore the optimal number of oocytes need to retrieve during controlled ovarian stimulation although verdicts are controversial. Rubio C and Verberg M F, et al. demonstrated that ovarian hyperreactivity may not only increase the risk of chromosomal abnormalities in embryos but reduce the quality of embryos and the rate of high-quality embryos^16,17^. However, other prospective studies concluded that the live birth rate (LBR) is impervious to the ovarian hyperreactivity despite its negative role in fertility rate and the CLBR accompanied by the number of available embryos remains to increase, which can also be reflected in our investigation. Based on our analysis, the fertilization rate and high-quality embryo rate of IVF/ICSI in the group ≤ 35 years of age were significantly lower than those ≥ 36 years of age while the clinical pregnancy rate, embryo implantation rate, live birth rate and CLBR were higher. Consequently, for elderly patients, embryos accumulation by multiple ovulation induction can raise the rate of normal karyotype embryo, which then increase the chance of a live birth accordingly. Notably, the stratification of participants primarily relies on the risk of aneuploidy in our study, which is known to increase significantly beyond 35–36 years. Future researches can further speculate the potential correlation between age, the number of oocytes retrieved and the likelihood of ploidy particularly in the absence of preimplantation genetic testing for aneuploidy (PGT-A), which may also be responsible for the decline in cumulative live birth rate after the age of 35.

As is well known, the number of oocytes retrieved and the age of patients make up the most important independent factors affecting the CLBR. Consistent with other studies, we confirmed the independent property of them after controlling confounding factors including “fertilization rate”, “high-quality embryo rate” and “fertilization type”. The correlation analysis between the number of oocytes retrieved and the CLBR in each age stratum revealed that the number of oocytes retrieved had potential predictive value in the CLBR. For patients ≤ 35 years of age, the CLBR can reach more than 60% as long as the number of oocytes retrieved is between 6 and 8; but patients aged 36–37 years of age needed 9–13 oocytes to achieve a CLBR aboving 60%. What's worse, for patients aged 38–39 years of age, the CLBR of more than 60% can be accomplished only when the oocytes numbers are greater than 14 while the CLBR in patients ≥ 40 years of age can hardly reach 60% even if the number is more than 14.

The most significant limitation of this study lies in its retrospective design, which is associated with inherent bias in the research itself that affects our outcomes. In addition, the long study period and clinical operational errors may inevitably be confounding factors influencing experimental results. Although we tried our best to minimize selection bias and control for knowable confounders through strict inclusion criteria, other undiscovered confounders cannot be completely eliminated. At the same time, during the period from 2013 to 2017 of the case data collected in this study, our reproduative center had not included AMH test and the antral follicle count as the routine testing program and we can not obtain these values, so we could only use basic endocrine value to represent the difference between ovarian function, which will be further improved and optimized in our subsequent study. In addition, the protocol of ovulation induction in fresh embryo transfer cycles can hardly be reconcile which varied in different groups, the older group mainly adopted the long scheme while the younger tend to receive the short scheme, leading to the discrepancy in the number of oocytes retrieved, and thus inevitably affecting the CLBR. Furthermore, the endometrial preparation pattern of freezing–thawing cycles is also inconsistent in the whole population. However, it is worth clarifying that no matter which preparation has an equivalent effect on the clinical outcome of freeze-thawing embryos^18^.

## Conclusion

The age and number of oocytes retrieved are two independent vital factors affecting cumulative live birth rate. The cumulative live birth rate in patients ≤ 35 years old can achieve over 60% as long as the number of oocytes obtained is more than 6, indicating that practice of controlling the stimulation dose to ensure the live birth rate and reduce the occurrence of OHSS is feasible for them. However, the elderly patients, especially those ≥ 38 years old, need to retrieve more than 14 oocytes to obtain higher live birth rate. Therefore, it is of great necessity to receive additional stimulation cycles and accumulation of embryos.

## Materials and methods

### Study population

17,931 women undergoing their first IVF/ICSI cycle in the Centre for Reproductive Medicine of the Sir Run Run Shaw Hospital of Zhejiang University between January 2013 and December 2017 were retrospectively analyzed in the database. This study was approved by the institutional review board of our hospital. All methods were performed in accordance with the relevant guidelines and regulations. All patients gave written informed consent.

**Patients’ eligibility criteria**: first IVF/ICSI cycle; live birth obtained from fresh or frozen embryo transplantation; no live birth but used all embryos.

**Patients’ exclusion criteria:** non-first IVF/ICSI cycle; zero oocyte retrieved; cycles with preimplantation genetic test; cycles with medical freezing oocytes; no live birth but still have frozen embryos.

- 1. For the age of patients, women were categorized in two groups: Group A (total 14,567 cycles): women ≤ 35 years of age; Group B (total 3364 cycles): women ≥ 36 years of age. Group B were further divided into into three age strata: 36–37 years (1361 cycles), 38–39 years (895 cycles) and ≥ 40 years (1108 cycles).
- 2. For the number of retrieved oocytes, women were categorized into four groups: a ≤ 5, b: 6–9; c: 10–14; d: ≥ 15.

### Treatment protocols (IVF/ICSI-ET)

All eligible patients were treated with recombinant and/or urinary gonadotrophins (rFSH/hMG) in ovarian stimulation protocols followed by IVF or ICSI. The choice of ovarian stimulation protocols and gonadotrophin dose depends on age, ovarian reverse function and number of basal follicles. Follicular development was monitored by ultrasound scanning, and final oocyte maturation was induced by injection of human chorionic gonadotrophin (hCG) when at least three follicles of 18 mm in diameter were observed. Oocytes retrieval was performed 36 h after hCG administration under the guidance of ultrasound.

Fresh embryo transfer was carried out under ultrasound guidance three or five days after oocyte retrieval. For luteal phase support, intramuscular injection progesterone (60 mg qd) combined with oral progesterone (20 mg qd) was started the day after oocyte retrieval, reduced to intramuscular injection progesterone (40 mg qd) combined with oral progesterone (20 mg qd) after a positive beta-hCG and until 8 weeks of pregnancy.

### Selection criteria for embryo transfer and freezing

Embryo quality was assessed according to the number of blastomeres and percentage of fragmentation as previously reported. An embryo with 6–12 blastomeres and grades 1 and 2 was defined as good quality^15^. Supernumerary Day 3embryos with at least four blastomeres and ≤ 35% fragmentation were selected for cryopreservation with vitrification protocols and transfer.

### Frozen-thawed embryo transfer cycle

Embryos were evaluated for morphological thaw survival immediately after warming. Embryos with at least 50% of their cells remain intact were considered as survival and able to be transferred. Three main types of clinical protocols were used for endometrial preparation: the natural cycle, hormone replacement cycle, or human menopausal gonadotropin (HMG)-stimulated cycles^15^. The type of preparation for the FET cycle was based on the menstrual cycle pattern of the patient.

### Main clinical outcome measures

The cumulative live birth rate (CLBR), which referred to the delivery of a live born in the fresh or in the subsequent frozen-thawed cycles in relation to age category, served as the primary treatment reference indicators. Only the first delivery was considered in the analysis for the CLBR. The clinical pregnancy rate, implantation rate, live birth rate, multiple birth rate and preterm birth rate were also analyzed in the two age-groups.

### Statistical analysis

Patients’ baseline characteristics and ovarian stimulation characteristics of the IVF/ICSI treatment were analyzed by age strata. Data analysis were performed using Statistical Package for Social Sciences version 22.0. Continuous data were compared using the Student’s t-test and presented as the mean value ± SD. Categorical data were analyzed with the Chi-Square test, described by number of cases including numerator and denominator, percentages, odds ratio (OR) and confidence interval (CI). To assess the association of cumulative live birth rate with the number of oocyte retrieved and age after adjustment for relevant confounders, a multivariate logistic regression using binary logistic regression was performed. The potential predictors considered for the analysis were duration of stimulation, duration of infertility, endometrial thickness, insemination type, fertilization rate, and good quality embryo rate, the age and number of oocytes retrieved. The regression model results were presented as an odds ratio (OR) with SE and 95% confidence interval (CI). The Hosmer–Lemeshow goodness-of-fit test assessed the goodness-of-fit of the regression models. Statistical significance was defined as a two-tailed p-value of 0.05.

### Ethics declarations

The study was approved by the Ethics Committee in Run Run Shaw Hospital of Zhejiang University.

### Consent to participate

The patients/participants provided their written informed consent to participate in this study.

## Data Availability

The original contributions presented in the study are included in the article. Further inquiries can be directed to the corresponding authors.
